# HIV-1 Transcriptional Activator Tat Inhibits *IL2* Expression by Preventing the Presence of Pol II on the *IL2* Promoter

**DOI:** 10.3390/biom13060881

**Published:** 2023-05-24

**Authors:** Spyridoula Anastasopoulou, Tassos Georgakopoulos, Athanasia Mouzaki

**Affiliations:** Laboratory of Immunohematology, Division of Hematology, Department of Internal Medicine, Medical School, University of Patras, GR-26500 Patras, Greece; s_anastasopoulou@upatras.gr (S.A.); georgako@upatras.gr (T.G.)

**Keywords:** HIV-1, *IL2*, Tat, Pol II, NFAT2, J-LAT, transcription factors

## Abstract

HIV-1 infection leads to a gradual loss of T helper cells, chronic immune activation, and eventual immune system breakdown. HIV-1 causes deregulation of the expression of IL-2, a cytokine important for T helper cell growth and survival, which is downregulated in HIV-1 patients. The present study addresses the regulation of *IL2* expression via HIV-1 Tat transcriptional activator. We used J-LAT cells, a T cell line that serves as a latency model for studies of HIV-1 expression in T cells, and as controls a T cell line lacking HIV-1 elements and a T cell line with a stably integrated copy of the HIV-1-LTR promoter. We show that endogenously expressed Tat inhibits *IL2* transcription in J-Lat cells via its presence in the ARRE-1/2 elements of the *IL2* promoter and that the inhibition of *IL2* expression is mediated by Tat inhibiting Pol II activity at the *IL2* promoter, which is mediated by preventing the presence of Pol II at the ARRE-1/2 elements. Overall, Tat is present at the *IL2* promoter, apart from its cognate HIV-1 LTR target. This supports our current knowledge of how HIV-1 affects the host transcriptional machinery and reflects the potential of Tat to disrupt transcriptional regulation of host genes to manipulate cell responses.

## 1. Introduction

HIV infection is characterized by chronic activation of the immune system, uncontrolled viral replication, and a gradual decline in CD4^+^ T helper (Th) cells. The introduction of highly active antiretroviral therapy (HAART) immediately after diagnosis has resulted in chronic rather than fatal infection. Although the life expectancy of HIV-1-infected individuals who respond well to the drugs has improved dramatically with the long-term use of HAART [1], studies show that the virus is not completely eliminated in the plasma of patients and viremia is observed [2]. This inability to eliminate the virus is attributed to cells that serve as viral reservoirs and are responsible for the latency of infection [3,4]. The exact molecular mechanisms underlying viral latency are still unknown [5,6,7]. The viral reservoir consists of cells harboring transcriptionally integrated silent proviruses capable of producing infectious virions after treatment interruption. Once HIV is integrated into the host genome, its transcription is regulated by a complex of cis and trans factors [8]. Many studies suggest that repressive chromatin states, DNA methylation, and post-transcriptional changes in histones and non-histone proteins, as well as host transcription factors and the HIV-1 Tat protein, determine the transition to stable latency [7,8]. The presence of this latent reservoir is associated with chronic immune activation and persistent inflammation [9].

One of the most important effects of chronic HIV-1 infection is the dysregulation of the expression of a number of cytokines such as IL-2, IL-17, IFN-γ, and others [10]. The expression of IL-2 plays a critical role in infection, as it is the first cytokine secreted by naive Th cells after their activation by antigens [11], and it has been found to be downregulated in HIV-1 patients [12,13]. This was confirmed by in vitro studies using Jurkat T cells, which can express the stably integrated HIV-1 *Tat* [14,15] or the entire stably integrated virus [16] in their genome and, in this respect, behave exactly like CD4^+^CD25^-^ primary Th cells latently infected with HIV-1. Production of IL-2 is a measure of immunological activation of T cells via the downstream T cell receptor (TCR) pathway. Therefore, impaired IL-2 expression is associated with deficient development and proliferation of lymphocytes, monocytes, and macrophages, as well as inefficient viral replication and spread. For this reason, IL-2 is also important for latency. Its expression in acutely or latently infected T cells is impaired after in vitro stimulation with mitogens at the transcriptional level, albeit in a completely opposite manner. *IL2* mRNA levels are increased in acutely infected stimulated cells, whereas they are decreased in latently infected stimulated cells compared with stimulated uninfected cells [16]. Exogenous addition of IL-2 to patient T cells promotes their proliferation and protects them from apoptosis [17]. In addition, immunotherapy with IL-2 has been shown to increase Th cell numbers [18], reduce the pool of latently infected resting Th cells [19], and elicit HIV-1-specific T cell responses [20]. For these reasons, production of IL-2 by patients’ T cells is essential for proper immune function.

*IL2* gene activation occurs after binding of specific transcription factors to its promoter [21,22]. The transcription factors NF-κΒ and NFAT recognize purine-rich binding sites in the *IL2* promoter and play a central role in the signaling pathway that regulates *IL2* expression [21,23]. HIV-1 Tat protein is another transcription factor that affects *IL2* transcription [14,15,24].

The promoter region of *IL2*, which spans nucleotides −292 to −273 (ARRE-2 sequence) [22], is similar to the region of HIV-1-LTR, which spans nucleotides −279 to −250 (RATS sequence) [25]. The elements ARRE-2 and RATS are involved in suppressing the expression of the *IL2* gene and HIV-1, respectively [25,26,27]. Several transcription factors associated with *IL2* expression, including NF-κB and NFAT, are also involved in transcriptional activation of the HIV-1 genome via binding to the LTR viral sequence [24,28,29]. Binding of NF-κB to the LTR of HIV-1 stimulates its transcription, whereas NFAT appears to bind to the same NF-κB core element and not to the putative NFAT site (−255 to −217) in the LTR [28,29]. Many studies suggest that there is an interplay between host factors and the HIV-1 Tat protein in the regulation of HIV-1 [8,24,29,30].

Tat, a small RNA-binding protein, is the first protein expressed after HIV-1 infection and is required for efficient transcription and viral replication [24]. It is involved in apoptotic and survival mechanisms, alteration of T-cell proliferation, and expression of various cytokine genes [24]. The mechanism by which Tat regulates most cellular genes remains to be elucidated. Since Tat does not have a DNA-binding domain, many studies suggest that its activity is exerted on the expression of host genes either by modulating transcription through binding to TAR-like sequences in the 5′-untranslated region of nascent RNA in the promoter regions of genes or by interacting with transcription-regulating host proteins that form a Tat complex [24,31]. Tat has been reported to interact with various host proteins such as transcription factor Sp1, cyclin T, nuclear RNA polymerase II, and others [29,32,33]. The transcription factor NFAT1 also interacts with the HIV-1 Tat protein, and the two factors modulate each other’s activity [34]. Tat also activates NF-κΒ through its interaction with IκB-α and p65 [35].

Previous studies have shown that transient expression of HIV-1 Tat acts mainly by enhancing *IL2* promoter activity [36,37,38]. However, cells stably expressing Tat show a decrease in *IL2* expression [14,15,16]. From this point of view, transiently expressed Tat behaves analogously to cells acutely or transiently infected with the whole HIV-1 genome, whereas stably expressed Tat behaves similarly to a stably integrated virus [14,15,16]. These contrasting results in the regulation of *IL2* may reflect differences in gene regulation between acutely HIV-1 infected and latently infected cells [16].

The present study focuses on the impairment of *IL2* expression by HIV-1 Tat transcriptional activator expressed in J-Lat cells, a CD4^+^CD25^−^ Th cell line that serves as a latency model for studies of HIV-1 expression in Th cells. Because HIV-1 Nef protein has also been reported to affect *IL2* expression, J-Lat cells, which have the complete HIV-1 virus integrated into their genome but without the Nef gene, are an ideal model to study the effect of Tat on *IL2* expression in the absence of Nef [39] and avoid the artificial results caused by transient overexpression of Tat. We show that endogenously expressed Tat in induced latently infected J-Lat cells exerts its inhibitory effect on *IL2* transcription through its presence in the ARRE-1 and ARRE-2 elements of the *IL2* promoter and by preventing the presence of Pol II in the same elements.

## 2. Materials and Methods

### 2.1. Cells and Cultures

Jurkat (American type Culture Collection), Jurkat-Lat 6.3 (J-Lat) (NIH AIDS Reagent Program catalog number #9849), and Jurkat-LTRG (J-LTRG) (NIH AIDS Reagent Program catalog number #11587) T cell lines were used. Jurkat cells are derived from human T-cell acute lymphoblastic leukemia. J-Lat cells are Jurkat-derived cells latently infected with the packaged retroviral construct HIV-R7/E-/GFP. This is a full-length HIV-1 genome with a non-functional Env gene due to a frame shift and the green fluorescent protein GFP gene in place of the Nef gene [40]. J-LTRG cells, also derived from Jurkat cells, contain a stably integrated copy of the HIV-1 LTR promoter linked to the GFP reporter as a reporter for HIV-1 LTR expression [41,42]. In the absence of HIV-1 Tat expression, the cells exhibit extremely low eGFP expression.

All cell lines were cultured in RPMI-1640 medium supplemented with 10% heat-inactivated fetal bovine serum (FBS), 2 mM L-glutamine, penicillin (100 U/mL), and 50 μM 2-mercaptoethanol (CM) at a concentration of 10^6^ cells/mL in a humidified incubator at 37 °C and 5% CO_2_. When required, cells were stimulated with the mitogens ionomycin (2 μM) and phorbol myristate acetate (20  ng/mL) (P/I) for 6 h (peak *IL2* expression [26]).

### 2.2. Quantitative Real Time PCR (qPCR)

Total RNA was isolated using the standard method of Trizol according to the manufacturer’s instructions (Gibco). RNA yield and purity were determined by measuring absorbance at 260/280 nm on a Quawell microvolume spectrophotometer Q3000 (Quawell Technology). Total RNA (100 ng per reaction) was used for cDNA synthesis using M-MLV reverse transcriptase (200 U/μL) (Sigma-Aldrich, St. Louis, MI, USA) in 10× M-MLV reverse transcriptase buffer, 40 U/μL RNase inhibitor (Thermo Fisher Scientific-Invitrogen), 1 mM each dNTPs (Sigma-Aldrich), and 2.5 µM random hexanucleotide primers (Sigma-Aldrich). qPCR mRNA analysis was performed on an Mx3000P_TM_ Quantitative PCR System Thermocycler (Stratagene, San Diego, MA, USA) using SYBR-green fluorescence quantification technology (KAPA SYBR FAST qPCR Kit, Kapa Biosystems, Wilmington, MA, USA). PCR conditions were 95 °C for 15 min, followed by 36 cycles of 95 °C for 30 s to denature cDNA, 58 °C for 30 s for annealing, and 72 °C for 30 s for extension. The results were analyzed using MxPro^TM^ software (Stratagene). Expression of the β-actin gene served as a normalizer. All measurements were performed in triplicate. The primers used for qPCR (Eurofins Genomics/Oligo Analysis Tool) were:

*Tat*—5′-AGGAAGTCAGCCTAAAACTGC-3′ and 5′-GCTCTTCGTCGCTGTCTCC-3′, yielding a PCR product of 132 bp;

GFP—5′-GGTGATACCCTTGTTAATAG-3′ and 5′-AGGTAATGGTTGTCTGGTAAA-3, yielding a product of 176 bp;

*IL2*—5′-TCACCAGGATGCTCACATTTAAGT-3′ and 5′-GAGGTTTGAGTTCTTCTTCTTCTACAC-3′, yielding a PCR product of 127 bp;

*β-actin*—5′-TTGGCAATGAGCGGTTCC-3′ and 5′-AGCACTGTGTTGGCGTAC-3′, yielding a PCR product of 137 bp.

### 2.3. HIV-1 Tat siRNA Transfection

J-Lat cells were transfected with siRNA oligonucleotides using Lipofectamine LTX DNA transfection reagents (Invitrogen, Life Technologies) according to the manufacturer’s instructions. Cells were cultured in CM for 24 h (10^6^ cells per experimental point). The culture medium was changed 30 min before addition of the transfection reagent. Cells were incubated for 18–24 h in the presence of 10 nM of:

RNA oligo A [5′-GGAGCCAGUAGAUCCUAGA)TT-3′] or

RNA oligo B [5′-GCUUGUACCAAUUGCUAUU)TT-3′] (Eurofins Genomics/siRNA Design Tool).

After 24 h, the culture medium was changed, and 6 h later, the cells were cultured with P/I for an additional 6 h. They were then subjected to qPCR analysis and Western immunoblotting, as indicated in the results.

### 2.4. Western Immunoblotting

Whole-cell extracts (10^6^ cells per experimental point) were prepared using RIPA buffer (50 mM Tris-HCl pH 7.4, 150 mM NaCl, 1% Triton X-100, 1% sodium deoxycholate, 0.1% SDS, and 1 mM EDTA) supplemented with protease inhibitors (Sigma-Aldrich). Proteins were separated using SDS-PAGE and electrophoretically transferred to nitrocellulose membranes. Membranes were incubated with a rabbit polyclonal antibody to HIV-1 Tat (ab43014, Abcam, Cambridge, UK) and a rabbit monoclonal antibody to β-actin (Cell Signaling Technology #4970, Danvers, MA, USA), which served as a loading control. HRP-conjugated goat anti-rabbit IgG (sc-2004, Santa Cruz, CA, USA) was used as a secondary antibody. Protein levels were visualized using the ECL LumiGLO detection kit (Upstate, Biotechnology UBI).

### 2.5. Co-Immunoprecipitation

J-Lat cells were cultured in the presence (P/I) or absence (CM) of mitogens for 6 h. Whole protein extracts were then digested with RIPA buffer (50 mM Tris-HCl pH 7.4, 150 mM NaCl, 1% Triton X-100, 1% sodium deoxycholate, 0.1% SDS, and 1 mM EDTA) supplemented with protease inhibitors (Sigma-Aldrich). Extracts before immunoprecipitation were used as controls to ensure that all samples contained the same starting material (input). Extracts were subjected to immunoprecipitation with a rabbit polyclonal anti-Tat antibody (ab43014, Abcam) (0.1 μg/mL) using magnetic protein G beads (Dynabeads, Invitrogen). Protein–protein interactions between the transcription factors NFAT2 and Tip60 and HIV-1 Tat were examined using Western immunoblotting with a mouse monoclonal antibody to NFAT2 (sc-7294, Santa Cruz) and a mouse monoclonal antibody to Tip60 (sc-166323, Santa Cruz). An extract of J-Lat cells expressing NFAT and Tip60 without antibody (-Ab) was used as a negative control. A goat anti-rabbit IgG HRP-conjugated antibody (sc-2004, Santa Cruz) and a goat anti-mouse IgG HRP-conjugated antibody (sc-2005, Santa Cruz) were used as secondary antibodies.

### 2.6. Chromatin Immunoprecipitation (ChIP) Assays

For ChIP assays, Jurkat, Jurkat-Lat, and Jurkat-LTRG cells (10^7^ cells per experimental point) were cultured in CM or P/I for 6 h. Cells were fixed in 1.1% formaldehyde for 10 min and then quenched with 125 mM glycine for 5 min. They were then lysed and sonicated to generate 200–500 bp DNA fragments. ChIP assays were performed as described [26]. All reactions were performed in 1 mL sample tubes using 10 µg of isolated chromatin with 40 µL of protein G beads (Dynabeads, Invitrogen) and 5 µg of the corresponding antibody for each ChIP reaction. The antibodies used were anti-HIV-1 Tat (ab43014, Abcam) and mouse monoclonal anti-Pol II (sc-9001, Santa Cruz). Immunoprecipitated DNA from each cell line was analyzed for detection of specific sites with qPCR using the KAPA SYBR FAST qPCR kit (Kapa Biosystems) and the Mx3000P_TM_ Quantitative PCR System Thermocycler (Stratagene). The sequences of the ChIP primers used for the different genomic regions in qPCR (Eurofins Genomics/Oligo Analysis Tool) were:

ARRE-1/TATA—5′-TCTTTGGGGGTTTAAAGAAATTC-3′ and 5′-AGGAGTTGAGGTTACTGTGAG-3′, yielding a PCR product of 217 bp;

ARRE-2—5′-CTTGCTGTTGTCCACCAC-3′ and 5′-TGGATGTAGGTGAAATCCC-3′, yielding a PCR product of 201 bp;

RATS region of HIV-1-LTR—5′-CCTTTGGATGGTGCTACAAGC-3′ and 5′-GATGCAGCTCTCGGGCCA-3′, yielding a PCR product of 137 bp;

TATA region of the β2m promoter—5′-CGCCGATGTACAGACAGCAAA-3′ and 5′-TGCTGTCAGCTTCAGGAATG-3′, yielding a PCR product of 230 bp;

TATA region of the β-actin gene promoter—5′-CGGCGAAGCCGGTGAG-3′ and 5′-CTGGCGGGGGCTACGC-3′, yielding a PCR product of 175 bp.

The optimized PCR conditions were 95 °C for 10 min, followed by 40 cycles of 95 °C for 30 s and 60 °C for 30 s. The results represent the ChIP signal as a fold increase in signal compared with the background signal. In this normalization method, the fold enrichment is calculated (2^−DDCt^) and divided by the non-specific adjustment (Ct IP)—(Ct mock). Ct is the cycle at which the threshold line is crossed.

### 2.7. Statistical Analysis

Data are given as means (SD or SE) of three independent experiments. Statistical probabilities were evaluated using Student’s *t* test. The statistical significance level was set at *p* < 0.05. Data analysis and graphical representation were performed using GraphPad Prism v.9.0 software.

## 3. Results

### 3.1. Decreased IL2 mRNA Expression in Induced J-Lat Cells Due to Tat Inhibition

To investigate the role of Tat-HIV-1 in *IL2* transcription and to decipher the underlying mechanism, we determined *IL2* mRNA levels in J-Lat cells using qPCR. J-Lat cells were cultured under non-induced conditions (CM) and then induced with P/I for 6 h. The genetic background of J-Lat cells allowed us to avoid the effects of exogenous expression of Tat protein [36,37,38] (Supplementary Figure S1) and the effects of Nef protein on *IL2* mRNA expression [39].

**Figure 1:**
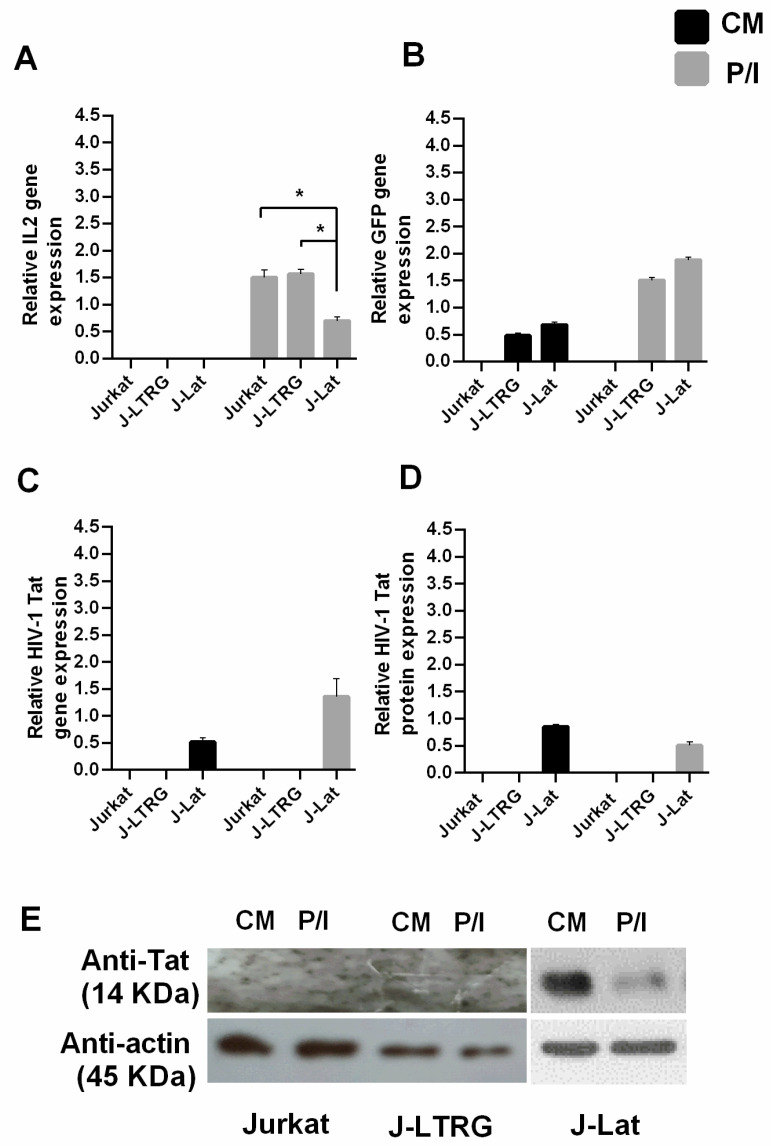
**Induced J-Lat cells express lower levels of *IL2* mRNA compared with induced Jurkat and J-LTRG cells.** (**A**) Relative *IL2* mRNA levels measured using qPCR in the T cell lines Jurkat, J-LTRG (carries HIV-1 LTR), and J-Lat (carries a noninfectious copy of HIV-1) cultured in CM or after induction with P/I for 6 h. (**B**) Relative mRNA synthesis of the GFP reporter gene. (**C**) Relative mRNA expression of the HIV-1 *Tat* gene. (**D**) Relative HIV-1 Tat protein expression in Jurkat, J-LTRG, and J-Lat cells measured using Western immunoblotting with specific antibodies to HIV-1 Tat. (**E**) Tat expression in Jurkat, J-LTRG, and J-Lat cells using Western immunoblotting with specific anti-Tat antibodies. Results are shown as means of three independent experiments; error bars represent SE (* *p* < 0.05; Student’s *t* test). Actin was used as a protein loading control.

Jurkat and J-LTRG cells were cultured under the same conditions (CM and P/I) as controls. The mRNA levels of *IL2*, GFP, and Tat were measured using qPCR in all cell lines under both conditions. As expected, *IL2* mRNA was upregulated in all cell lines after induction (Figure 1A). Consistent with our current knowledge of the function of Tat [24] and LTR-HIV-1 expression controlled by host transcription factors such as Sp1 and NF-κB [43] in the presence of mitogens, induction of J-Lat and J-LTRG cells with P/I resulted in GFP mRNA production in both cell lines (Figure 1B) and increased levels of endogenously produced *Tat* mRNA in induced J-Lat cells compared with non-induced cells (Figure 1C) [40,44,45]. The presence of Tat protein in J-Lat cells was detected using Western immunoblotting (Figure 1D,E). The discrepancy between the increased mRNA levels of Tat and the lower protein levels in induced J-Lat cells could be due to an asymmetry between the viral mRNAs produced and the actual functional viral mRNAs leading to the production of Tat protein [45], and/or to the repressive effect that Rev [46] and Gag gene products of HIV-1 [47] exert on the expression of Tat protein without affecting its mRNA expression.

When we compared the *IL2* mRNA levels produced after induction between cell lines, we found that they were significantly lower in induced J-Lat cells than in induced Jurkat or J-LTRG cells (Figure 1A). The difference between J-Lat, J-LTRG, and Jurkat cells under induced conditions is the complete expression of the integrated HIV-1 genome in J-Lat cells without the Nef and Env genes, suggesting that the Tat protein is likely a repressor of *IL2* expression. This observation is consistent with previous studies showing the same effect on *IL2* mRNA levels in cells with HIV-1 Tat or the entire HIV-1 virus integrated into their genome [14,15,16] and with the known role of Tat as a transcriptional repressor [15,48].

**Figure 2:**
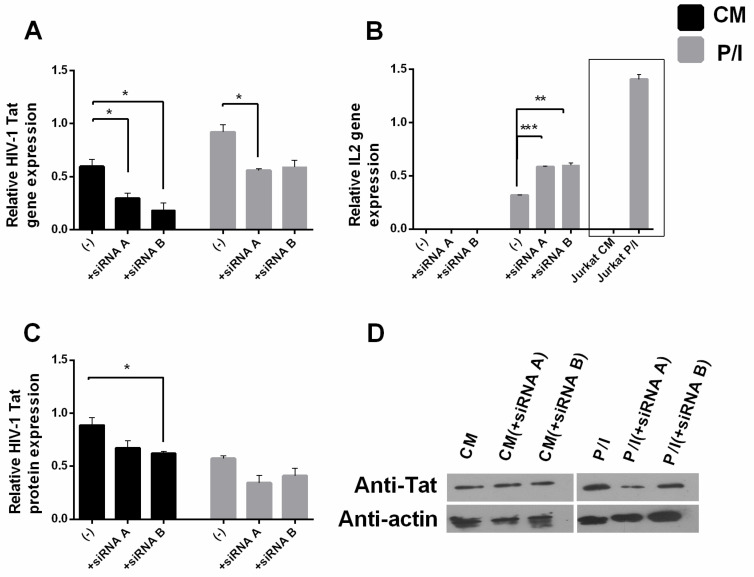
**Silencing of endogenous HIV-1 *Tat* results in the relief of *IL2* mRNA expression in induced J-Lat cells.** J-Lat cells were transfected with siRNA A and siRNA B and cultured in CM or with P/I for 6 h. Relative HIV-1 *Tat* (**A**) and *IL2* (**B**) mRNA levels in J-Lat cells. Additionally, shown in (**B**) for comparison are relative *IL2* mRNA levels in Jurkat cells cultured under the same conditions. (**C**) Relative HIV-1 Tat protein expression measured using Western immunoblotting with specific antibodies against HIV-1 Tat in J-Lat cells. (**D**) Representative Western blots of HIV-1 Tat protein expression in J-Lat cells. Results are presented as the result of the comparative quantification algorithm ΔΔCt. Results are presented as means of three independent experiments; error bars represent SE (* *p* < 0.05, ** *p* < 0.01, *** *p* < 0.001; Student’s *t* test). Actin was used as a protein loading control.

To investigate whether the HIV-1 Tat transcription factor per se was responsible for the low *IL2* mRNA levels in stimulated J-Lat cells, we silenced its endogenous expression with two different *Tat*-specific siRNA oligonucleotides, oligo A and B (Figure 2, see also M&M). After transfection, the cells were cultured ±P/I for 6 h. Tat protein reduction was detected using qPCR (Figure 2A) and Western blot analysis (Figure 2C,D). *IL2* mRNA expression was determined using qPCR. The results show that when Tat expression was inhibited in stimulated J-Lat cells, the expression of IL-2 mRNA was higher than that in induced J-Lat cells in which *Tat* was not silenced (Figure 2B), confirming that Tat can inhibit *IL2* expression independently of the presence of other viral genes. *IL2* mRNA in induced J-Lat cells in which *Tat* was silenced did not reach the level of mRNA expressed in induced Jurkat cells, because *Tat* was not completely silenced in these cells. Overall, these results demonstrate that endogenous expression of the HIV-1 Tat transcriptional activator in the context of a nearly intact viral genome, but in the absence of Nef and Env genes, does not allow complete transcriptional expression of the *IL2* gene after cell induction in T cells stably expressing Tat.

### 3.2. Tat Inhibits IL2 Expression by Preventing the Presence of Pol II on the IL2 Promoter

**Figure 3:**
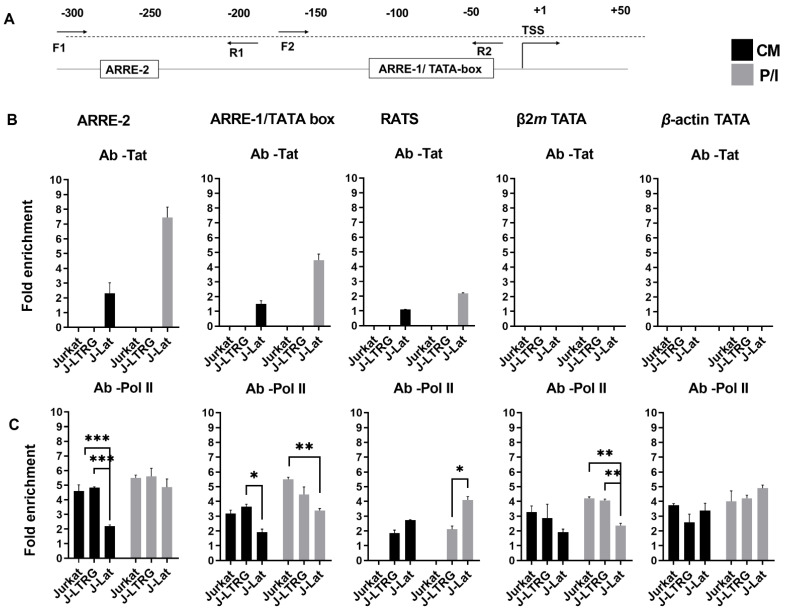
**Binding affinity of endogenously expressed Tat to *IL2* loci. ChIP assays in Jurkat, J-LTRG, and J-Lat cells.** (**A**) Schematic representation of the human *IL2* promoter showing the relative position of the ARRE-2 and ARRE-1/TATA box regulatory elements. The arrows represent the primer pairs used for each PCR amplification. (**B**,**C**) ChIP analysis in Jurkat, J-LTRG, and J-Lat cells cultured in CM or after induction with P/I for 6 h with qPCR. Binding to the *IL2* promoter regions ARRE-1/TATA and ARRE-2 and the RATS sequence of HIV-1 LTR. The binding affinity of the β2m TATA promoter region was used as a negative control. The β-actin TATA promoter was used as a control to check the specificity of the results. ChIP assays were performed with antibodies against Τat (**B**) and Pol II (**C**). Results represent ChIP signal as a fold increase in signal relative to background signal. Results are presented as means of three independent experiments; error bars represent SE (* *p* < 0.05, ** *p* < 0.01, *** *p* < 0.001; Student’s t test).

Although it has been proposed that the impairment of *IL2* mRNA levels in HIV-1-infected cells is due to the impairment of the TCR pathway [16], we hypothesized that endogenously expressed Tat may play a more direct role in *IL2* transcription. *IL2* transcriptional activation is known to be under the control of several host transcription factors that either enhance or repress it. The ARRE-2 DNA element of the *IL2* promoter, which is also an NFAT2 binding site, plays a central role in the pro-inductive repression of *IL2* transcription in naive CD4^+^CD25^−^ Th cells and the Jurkat cell line [26]. Since Tat has been described elsewhere as a transcriptional repressor [15,48] and is present in a complex that can bind to the ARRE-2 element [34], we investigated whether the lower *IL2* transcription in induced J-Lat cells compared to *IL2* mRNA levels in induced Jurkat and J-LTRG cells was consistent with the presence of the Tat protein in the *IL2* promoter in J-Lat cells. To investigate the physiological interactions between Tat and the *IL2* promoter in vivo, we performed ChIP analysis using chromatin from untransfected Jurkat, J-LTRG, and J-Lat cells cultured in the presence or absence of P/I. Cross-linked chromatin was subjected to immunoprecipitation with antibodies to Tat and human Pol II or a negative control antibody to IgG. The association of Tat and Pol II with the *IL2* promoter and HIV-1 LTR was detected with qPCR using specific primer sets (Figure 3A). We examined the presence of Tat at ARRE-2 and ARRE-1, the region of the *IL2* core promoter that includes the TATA box (which also contains an NFAT-binding sequence) and the RATS element of LTR (Figure 3B). The relative positions of ARRE-2 and ARRE-1 are shown in Figure 3A. The chromatin regions encompassing the promoters of the human β2m and actin genes and containing the TATA element were selected as negative controls for Tat binding (Figure 3B).

Because Tat is not expressed in Jurkat and J-LTRG cells and Jurkat cells also do not have HIV-1 LTR in their genome, we were unable to detect Tat in ARRE-2, ARRE-1, and RATS in either cell line under either CM or P/I conditions (Figure 3B). In J-Lat cells expressing Tat under both CM and P/I conditions (Figure 1C,D), Tat protein was detected in ARRE-2, ARRE-1, and RATS, but at unequal levels. More specifically, Tat levels decreased from ARRE-2 to ARRE-1 to RATS under both CM and P/I conditions. At the same time, compared with CM, Tat binding increased three-fold after induction (P/I) in ARRE-2 and ARRE-1 elements and two-fold in RATS (Figure 3B Ab-Tat). The above results indicate that Tat shows a higher preference for the ARRE-2 and ARRE-1 elements of the *IL2* promoter than for the RATS element of HIV-1 LTR in latent J-Lat cells under both CM and P/I conditions, which may also mark its interference with *IL2* transcription. This preference and its three-fold higher presence in ARRE-2 and ARRE-1 after cell induction is accompanied by a shorter *IL2* mRNA induction compared with the other two cell lines that do not express Tat (Figure 1A). It is interesting to note that when we performed the same ChIP assays in previously transfected Jurkat and J-LTRG cells with a pcDNA3-based plasmid overexpressing Tat (1-101 a.a.), we failed to detect the presence of exogenously added Tat on the *IL2* promoter (Supplementary Figure S2), which may explain the discrepancy in *IL2* mRNA expression between endogenous and exogenously added Tat in the literature [14,15,36,37,38], (Supplementary Figure S1). Control ChIP assays with the Tat antibody showed no presence of Tat at the β2m or actin gene promoters under all conditions tested and in all cell types, a result confirming the specificity of our experiments.

Since the ARRE-2 element is known to be involved in the transcriptional repression of *IL2* [26], we examined the effects of endogenous Tat expression on the presence of Pol II in the *IL2* promoter before and after induction in all cell lines. In Jurkat and J-LTRG cells, Pol II was found to interact with the ARRE-2 element in CM and P/I to an almost similar extent between cell lines. In both cell lines, Pol II also interacted with ARRE-1 in CM, although to a lesser extent than with ARRE-2 under the same conditions. In both cell types, the presence of Pol II in ARRE-1 increased at P/I, consistent with transcriptional activation of the *IL2* gene after induction, and nearly reached the level of presence in ARRE-2 under the same conditions (Figure 3C). In contrast, the presence of Pol II in J-Lat cells was lower than in the other two cell lines in both ARRE-2 and ARRE-1 at CM. These differences between Jurkat, J-LTRG, and J-Lat cells in the presence of Pol II in ARRE-2 and ARRE-1 at CM may be attributed to the simultaneous presence of Tat in both elements at CM in J-Lat cells, leading to the exclusion of Pol II from the ARRE-2 and ARRE-1. The presence of Pol II at the ARRE-2 and ARRE-1 elements under CM conditions in all cell lines may reflect its association with one or more factors that bind to these elements in a ready-to-use state [26]. After induction (P/I) of J-Lat cells, the presence of Pol II in ARRE-2 reached almost the same levels as in the other two cell lines. This was not the case in ARRE-1, where the presence of Pol II in P/I was ~60% of that in Jurkat cells and ~75% of that in J-LTRG cells. This may be analogous to the exclusion of Pol II from Tat in ARRE-1 at CM in J-Lat, because the concomitant presence of Tat in ARRE-1 under P/I conditions results in impairment of *IL2* transcription during transcriptional activation, possibly by impeding the function of Pol II.

Because of the absence of an HIV-LTR sequence in Jurkat cells, we could not detect binding of Pol II with the specific primer set for the RATS element under both CM and P/I conditions (Figure 3C). In J-LTRG cells, the binding of Pol II to the RATS element was almost identical under CM and P/I conditions, suggesting that the underlying transcription of the HIV-1 LTR region in the absence of transcriptionally active virus, and particularly in the absence of Tat is due to the leakiness of the LTR in CM and during induction (P/I) to the action of host transcription factors such as NF-κB and NFAT [28,29] (Figure 3C). This result is consistent with GFP expression in J-LTRG cells grown under CM and/or P/I conditions (Figure 1B).

Similarly, Pol II is present in the genomic region surrounding the TATA box of the β2m promoter under both CM and P/I conditions in Jurkat and J-LTRG cells. In contrast, the presence of Pol II in the β2m TATA was reduced by almost half in induced J-Lat cells compared with induced Jurkat and J-LTRG cells, consistent with the fact that the previously reported [49] transcriptional repression of β2m mRNA expression in cells latently infected with HIV-1 is a completely different effect from that observed for *IL2* mRNA expression because there is no concomitant presence of Tat in the β2m promoter. In contrast, binding of Pol II to the TATA element of the β-actin promoter (where Tat is also absent) is not affected under either non-induced or induced conditions in all cell lines. Taken together, our results confirm that the suppressive effect of Tat on *IL2* mRNA expression in cells latently infected HIV-1 is Tat-specific and is related to its presence at the *IL2* promoter and, in particular, at ARRE1 by completely preventing the concomitant presence of Pol II.

**Figure 4:**
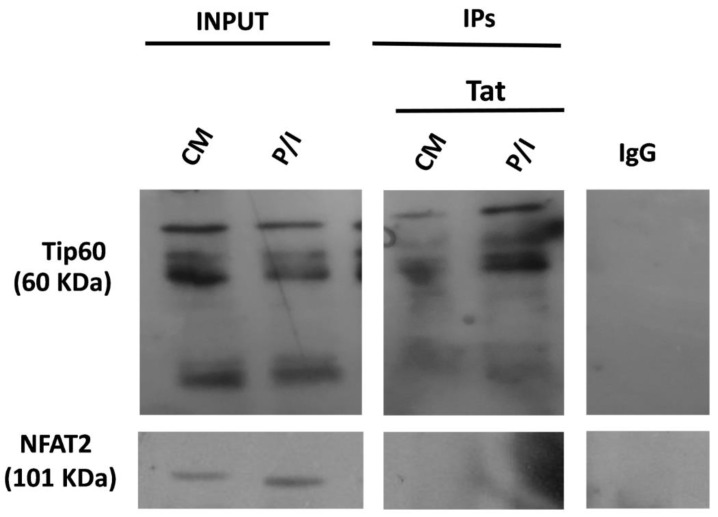
**Tat and NFAT2 do not physically interact in J-Lat cells**. J-Lat cells were cultured ±P/I for 6 h. Extracts before immunoprecipitation were used as controls to ensure that all samples contained the same starting material (input). Extracts were subjected to immunoprecipitation with an anti-Tat antibody. Immunoprecipitates were analyzed using Western immunoblotting with an anti-NFAT2 monoclonal antibody. An extract from J-Lat cells with IgG antibodies was used as a negative control. As a positive control, extracts from the same cells were immunoprecipitated with a human anti-Tat antibody to detect the interaction of Tat with Tip60. The bands shown in the gel represent the Tip60 isoforms. Tip60 isoforms were detected between 60 and 53 kDa.

Since Tat has no DNA-binding domain and its action on HIV-1 LTR requires the TAR element [24], its presence at the *IL2* promoter and subsequent hindrance of *IL2* transcriptional activation must be attributed to interactions of Tat with host transcription factors. Tat is known to interact with NFAT1 [34] and the p65 subunit of NF-κB [34], two factors involved in *IL2* transcriptional activation [21,22]. Since the interaction of Tat in both cases leads to an increase in the transcriptional activities of NFAT1 and NF-κB at many genes [34,35], we performed co-immunoprecipitation experiments to investigate whether Tat interacts with another NFAT member, NFAT2, to find a possible interaction to which we could attribute the inhibitory effect of endogenously expressed Tat on induced *IL2* transcription. We found that Tat did not interact with NFAT2 under either CM or P/I conditions. As a control for the accuracy of our assay, we demonstrated the known interaction between Tat and Tip60 protein [50] (Figure 4).

## 4. Discussion

**Figure 5:**
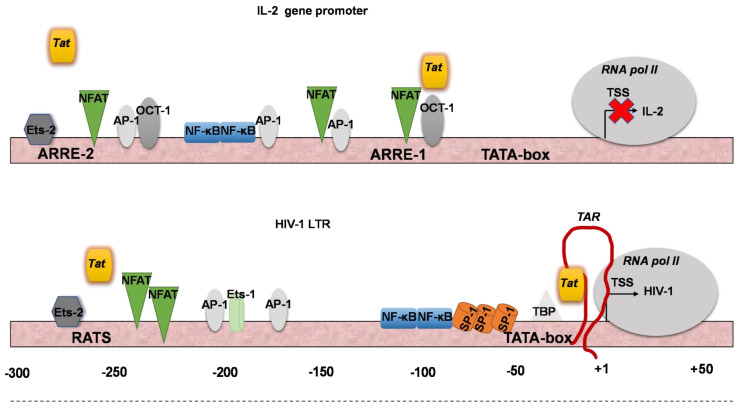
**Model of Tat activity on the *IL2* promoter**. Schematic representation of transcription factors binding to the regulatory elements of the *IL2* promoter ARRE-1/TATA and ARRE-2 and the RATS sequence of HIV-1 LTR.

Overall, our results show that Tat, when endogenously expressed in J-Lat cells, has a high presence at the *IL2* promoter, apart from its cognate HIV-1-LTR target. This not only supports our current knowledge of HIV-1 hijacking the host transcriptional machinery, but also reflects the potential of the Tat activator to exert its multiple functions by disrupting transcriptional regulation of many genes, such as cytokine genes, to manipulate cell responses (Figure 5). Since Tat lacks a DNA binding domain, its presence in the *IL2* promoter in the two distinct elements ARRE-1 and ARRE-2, which are several base pairs apart, must be due to its interactions with host transcription factors involved in *IL2* transcriptional regulation. Because the inhibitory effect on *IL2* transcription in latently infected J-Lat cells occurs during cell induction, these interactions could involve NF-κB and/or NFAT1 and other transcription factors, but not NFAT2, as shown here. Future studies will elucidate the nature, type, and exact number of these Tat interactions.

## Data Availability

All raw data are available upon request from the corresponding author.

## Supplementary Materials

The following supporting information can be downloaded at: https://www.mdpi.com/article/10.3390/biom13060881/s1, Figure S1: Effects of exogenously added Tat on mRNA expression of IL-2 in Jurkat, J-LTRG, and J-Lat cells; Figure S2: Binding affinity of exogenously expressed Tat to the IL-2 promoter in transfected Jurkat, J-LTRG, and J-Lat cells. Reference [51] is cited in the supplementary materials.
