# Supplementary material for: HIV-1 Transcriptional Activator Tat Inhibits IL2 Expression by Preventing the Presence of Pol II on the IL2 Promoter
Source: Biomolecules. 2023 May 24;13(6):881. doi: 10.3390/biom13060881 (PMC10296241; doi:10.3390/biom13060881)
Supplement: Supplementary file 1 [file biomolecules-13-00881-s001.zip › biomolecules-2377990-supplementary.pdf]

Manuscript ID: biomolecules-2377990

Section: Molecular Biology

Type of the Paper: Research Article

HIV-1 transcriptional activator Tat inhibits IL2 expression by preventing the presence of Pol II on the IL2 promoter

Spyridoula Anastasopoulou, Tassos Georgakopoulos and Athanasia Mouzaki

SUPPLEMENTARY MATERIAL

Method for cell transfections

Jurkat, J-LTRG, and J-Lat cells were transfected with a pcDNA-Tat 101 plasmid (a gift from Dr. J. Papamatheakis, IMBB, Crete, Greece) or an empty pcDNA3 vector (pcDNA3) using Lipofectamine LTX DNA transfection reagents (Invitrogen, Life Technologies) according to the manufacturer's instructions. Transfected cells were cultured in CM (containing 5% FBS) for 48 hours. When required, the cells were cultured with P/I for an additional 6 hours. They were then subjected to qPCR analysis and Western immunoblotting as indicated in the results below.

Exogenously expressed Tat, in contrast to endogenously expressed Tat, increases IL-2 transcription and is not present on the IL-2 promoter

Jurkat, J-LTRG, and J-Lat cells were transfected with a pCDNA3-based plasmid that resulted in expression of the full-length (1-101 a.a.) Tat protein (pcDNA-Tat 101 plasmid). Expression of Tat was detected by qPCR (Figure S1A) and Western immunoblotting (Figures S1D, E, F). When all cell lines were transfected with the

empty pcDNA3 vector, there were no differences in the expression of Tat, IL-2, or GFP mRNA compared with untransfected cells under the same culture conditions (Figure S1A, B, C). The absence of Tat expression in Jurkat and J-LTRG cells transfected with pcDNA3 was verified by Western immunoblotting (Figure S1D, E, F). Exogenously expressed Tat resulted in an increase in IL-2 mRNA in all cell lines cultured with P/I (Figure S1B) compared with untransfected cells or cells transfected with pcDNA3. In contrast, IL-2 mRNA is downregulated when Tat is endogenously expressed (Figure 1A, main manuscript). These results confirm previous studies showing opposite effects of endogenous and exogenous Tat expression on the regulation of IL-2 transcription [14-15, 36-38, main manuscript].

To further investigate these contrasting effects of endogenous and exogenous Tat expression on the regulation of IL-2 transcription, we performed ChIP assays to verify the presence of Tat on the IL-2 promoter (Figure S2). Exogenously expressed Tat was not detected on the ARRE2 or ARRE1/TATA elements of the IL-2 promoter in Jurkat and J-LTRG cells or the RATS element of HIV-LTR in J-LTRG cells (Figure S2A). The latter is consistent with the fact that there were no differences in expression and GFP mRNA in J-LTRG and J-Lat cells compared with untransfected cells or cells transfected with pcDNA3 under the same culture conditions (Figure S1C).

Taken together, these results suggest that the exogenously expressed Tat protein must have different properties than the endogenously expressed Tat. These differences may result in a differential effect on IL-2 mRNA levels, probably by Tat controlling or interacting with other factors responsible for IL-2 transcription in different ways or by sequestering factors that control or repress IL-2 transcription after mitogenic induction. This is consistent with the differential requirements for HIV-1 expression and subsequent integration into the host genome during acute HIV-1 infection compared to the latent state of the virus and is consistent with previously proposed explanations for these differences [14-16, main manuscript].

An additional reason for the differential effects of endogenously and exogenously expressed Tat on IL-2 transcription is that endogenously expressed Tat may form specific chromosomal contacts that exogenously expressed Tat cannot. These differences may explain the inability of exogenously expressed Tat to enhance transcription of endogenous HIV-1 LTR sequences compared with LTR reporter genes. Moreover, exogenously expressed Tat must compete with endogenously expressed Tat in J-LAT cells because the presence of Tat, at least on the ARRE1/TATA element, is impaired compared with untransfected cells or cells transfected with pcDNA3 (Figure S2B), possibly by enhanced dimerization in the ARRE1 element [Ref. S1].

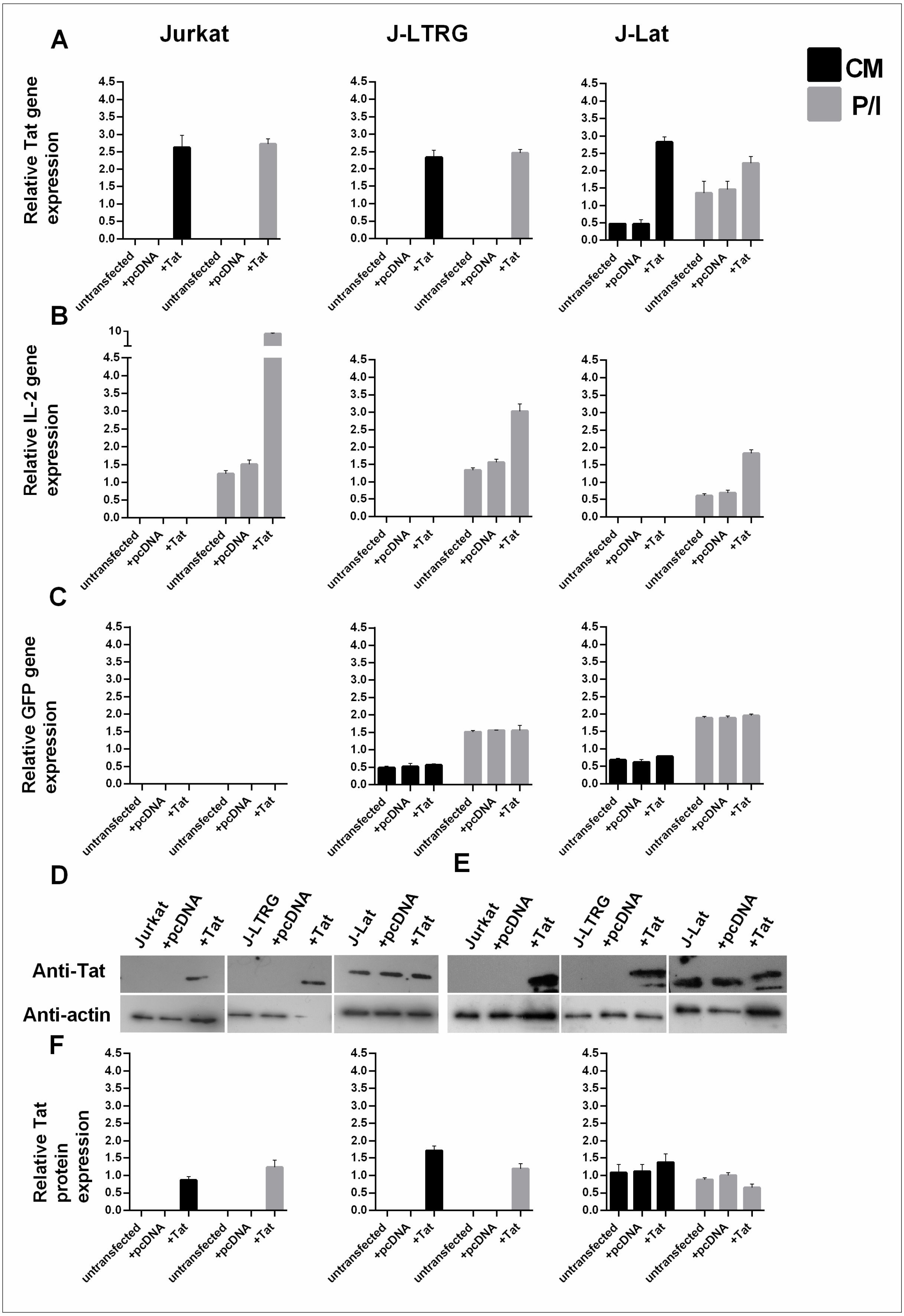
A
Jurkat
J-LTRG
J-Lat
CM
P/I
Relative Tat gene expression
untransfected
+pcDNA
+Tat
B
Relative IL-2 gene expression
C
Relative GFP gene expression
D
E
Jurkat
+pcDNA
+Tat
J-LTRG
J-Lat
Anti-Tat
Anti-actin
F
Relative Tat protein expression

Figure S1. Effects of exogenously added Tat on mRNA expression of IL-2 in Jurkat, J-LTRG, and J-Lat cells. The three columns show the results in transfected Jurkat, J-LTRG, and J-Lat cell lines cultured in CM or after 6-hour induction with P/I. (**A**) Relative mRNA expression of the HIV-1 Tat gene. (**B**). Relative IL-2 mRNA levels (**C**) Relative mRNA synthesis of GFP reporter gene. (**D**) Protein expression of HIV-1 Tat in untransfected cells or cells transfected with pcDNA3 as control or cells transfected with pcDNA-Tat 101 cultured in CM and (**E**) after induction with P/I for 6 hours, analyzed by Western immunoblotting with an anti-Tat monoclonal antibody. (**F**) Relative HIV-1 Tat protein expression in Jurkat, J-LTRG, and J-Lat cells measured by Western immunoblotting in D and E. Results are shown as means of three independent experiments; error bars represent SE (*$p < 0.05$; Student's *t* test). Actin was used as a protein loading control.

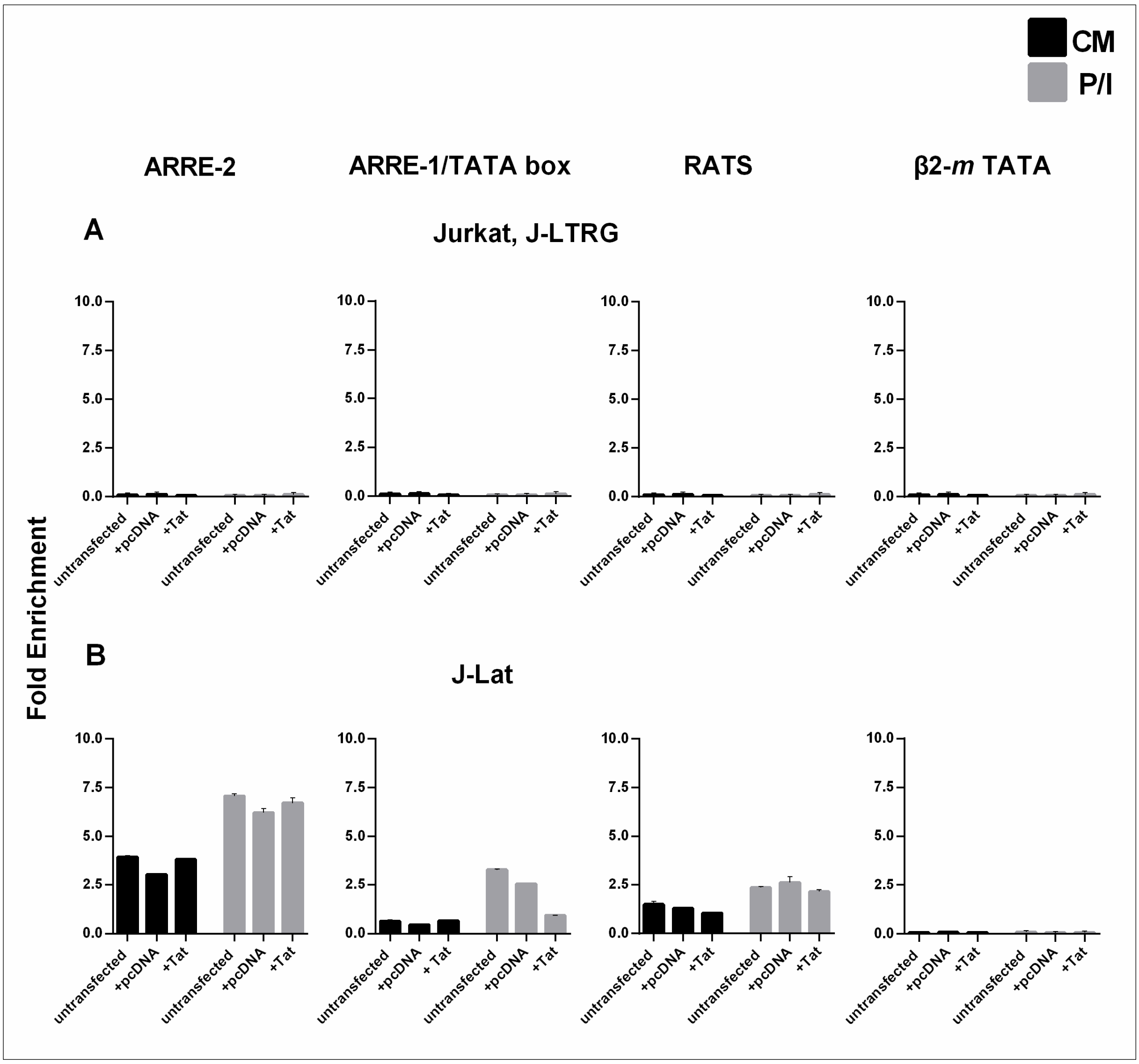

Figure S2. Binding affinity of exogenously expressed Tat to the IL-2 promoter in transfected Jurkat, J-LTRG, and J-Lat cells. ChIP analysis by qPCR in (**A**) Jurkat, J-

LTRG, and (**B**) J-Lat cells, cultured in CM or with P/I for 6 hours. The binding affinity of the β2-m TATA promoter region was used as a negative control. ChIP assays were performed with antibodies against Tat. Exogenously expressed HIV-1-Tat shows no binding affinity at the loci of the IL-2 promoter and the RATS sequence in all three cell lines. Results represent ChIP signal as a fold increase in signal relative to background signal. Results are shown as means of three independent experiments; error bars represent SE ($^*p < 0.05$, Student's t test).

Reference

S1. Frankel AD, Biancalana S, Hudson D. Activity of synthetic peptides from the Tat protein of human immunodeficiency virus type 1. Proc Natl Acad Sci U S A. 1989; 86(19):7397-7401.
